# Radon progeny measurements in a ventilated filter system to study respiratory-supported exposure

**DOI:** 10.1038/s41598-023-37697-7

**Published:** 2023-07-04

**Authors:** Franziska Papenfuß, Andreas Maier, Sonja Sternkopf, Claudia Fournier, Gerhard Kraft, Thomas Friedrich

**Affiliations:** 1 GSI Helmholtzzentrum für Schwerionenforschung GmbH, Planckstraße 1, Darmstadt, Germany; 2 Goethe Universität Frankfurt, Max-Von-Laue-Str. 1, Frankfurt, Germany

**Keywords:** Computational biophysics, Biological physics

## Abstract

Radon (^222^Rn) and its progeny are responsible for half of the annual dose from natural radiation and the most frequent cause for lung cancer induction after smoking. During inhalation, progeny nuclides accumulate in the respiratory tract while most of the radon gas is exhaled. The decay of progeny nuclides in the lung together with the high radiosensitivity of this tissue lead to equivalent doses implying a significant cancer risk. Here, we use gamma spectroscopy to measure the attachment of radon progeny on an air-ventilated filter system within a radon enriched atmosphere, mimicking the respiratory tract. A mathematical model was developed to describe the measured time-dependent activities of radon progeny on the filter system. We verified a linear relation between the ambient radon activity concentration during exposure and the amount of decay products on the filter system. The measured activities on the filters and its mathematical description are in good agreement. The developed experimental set-up can thus serve to further investigate the deposition of radon progeny in the respiratory tract under varying conditions for determination of dose conversion factors in radiation protection, which we demonstrate by deriving dose estimations in mouse lung.

## Introduction

The radioactive noble gas ^222^Rn is part of the decay series of ubiquitous 238U in natural rocks and soil and known as the most frequent cause for lung cancer induction after smoking^1–4^. As ^222^Rn is an inert gas, it does not attach to aerosols and the walls of the respiratory tract. Therefore, most of it is directly exhaled and simulations show that only about 1% of the inhaled gas dissolves in blood from where it is distributed all around the body^5^. In contrast to ^222^Rn, its decay products are solids under normal exposure conditions so that they may attach to aerosols they get in contact with^6^. Directly after decay, the α-emitting decay products ^218^Po and ^214^Po are highly charged^7,8^ but get rapidly neutralized via mechanisms as electron scavenging, electron transfer and ion recombination^9–12^, so that most of them carry no charge at the end of their recoil path^13^. During inhalation the progeny radionuclides are deposited in different regions of the respiratory tract depending on their size and shape as well as the physiological properties of the respiratory tract as e.g. morphology and air-flow velocity in different regions^14^. Larger particles are more likely to be exhaled compared to smaller unattached particles that get nearly completely adsorbed. Once deposited in the respiratory tract, the radon progeny radionuclides decay there because the clearance processes (mucociliary transport, drainage system or blood-flow) are much slower than the half-life of all radon progeny until ^210^Pb^15^. Therefore, no efficient clearance from the respiratory tract occurs and the decay energy of the nuclides that is released until they decay to ^210^Pb is deposited in the respiratory tract.

The relevant part of the ^222^Rn decay chain is shown in the following^16^:

$$\begin{array}{*{20}c} {^{\bf 222} {\bf Rn}} \\ {3.8\,\, {\text{d}}} \\ \end{array} \mathop{\longrightarrow}\limits_{{\upalpha \,\,5.49 \,\,{\text{MeV}}}}^{}\begin{array}{*{20}c} {^{\bf 218} {\bf Po}} \\ {3.05 \,\,\min } \\ \end{array} \mathop{\longrightarrow}\limits_{{\upalpha \,\, 6.00\,\,{\text{ MeV}}}}^{}\begin{array}{*{20}c} {^{\bf 214} {\bf Pb}} \\ {26.8\,\, \min } \\ \end{array} \mathop{\longrightarrow}\limits_{{\upbeta , \upgamma }}^{}\begin{array}{*{20}c} {^{\bf 214} {\bf Bi}} \\ { 19.9\,\, \min } \\ \end{array} \mathop{\longrightarrow}\limits_{{\beta , \gamma }}^{}\begin{array}{*{20}c} {^{\bf 214} {\bf Po}} \\ {164 \,\, \upmu {\text{s}}} \\ \end{array} \mathop{\longrightarrow}\limits_{{\upalpha \,\, {\text{7.69 MeV}}}}^{}\begin{array}{*{20}c} {^{\bf 210} {\bf Pb}} \\ {22.3\,\,{\text{a}}} \\ \end{array} .$$

During decay, the α-emitting isotopes are responsible for most of the deposited energy while the β- and γ-emitting isotopes are responsible for about 10% of the cumulative decay energy. Moreover, α-particles with a high linear energy transfer (LET) exhibit a higher biological effectiveness than β-particles or γ-radiation. This is also represented by the higher radiation weighting factor of 20 for this radiation type, compared to a radiation weighting factor of 1 for β-particle or γ-radiation^17^, which needs to be taken into account when calculating the equivalent dose. In good approximation only α-emitting particles are accounted for the calculation of the absorbed dose. All decay products following ^210^Pb are neglected due to the long half-life of this nuclide^18^.

With each breath, further radon progeny radionuclides accumulate in the respiratory tract and deposit their decay energy there. Simulations show that following ^222^Rn exposure, the progeny radionuclides account for more than 95% of the effective dose (whole-body dose, considering tissue weighting factors), whereby the ^222^Rn gas itself accounts for less than 5%. In addition, the lung equivalent dose makes up for over 95% of the effective dose^18^, which explains why radon and its progeny are classified as carcinogenic for the lung^19^. Thereby the lung is defined as the part of the respiratory tract starting from the trachea^14^.

Nowadays, detailed theoretical models on the deposition of radon progeny in the respiratory tract exist to calculate the dose-distribution and the corresponding risk^20,21^. They are validated by some experimental data on humans for different exposure scenarios^14,22^. But still no experimental set-up exists in which parameters such as breathing rate, geometry and air-flow velocity in the respiratory tract as well as the radon concentration and particle size distribution in air can be controlled to experimentally investigate the distribution of radon progeny in the respiratory tract. Therefore, our aim is to build an experimental set-up in which a simple mechanical surrogate of the respiratory tract is exposed in a well-defined adjustable atmosphere. This would allow to systematically test the theoretical models with a high number of experiments under the same, controllable conditions, which are not affected by e.g. the individual morphology of the respiratory tract. In a next step, readjustments of model parameters could be introduced to reflect individual variations.

This manuscript presents a first step to such a mechanical set-up. Here, radon containing air is pumped through a filter system, on which progeny radionuclides get deposited like they do in the respiratory tract. First measurements were conducted under well-defined exposure conditions where only the radon activity concentration and relative humidity in the exposure atmosphere were varied, whereas the other parameters were kept constant. This allowed us to develop a mathematical model to consistently describe the time-dependent distribution of radon and its progeny in the overall experimental set-up and especially on the ventilated filter system.

During the experiment, the filter system adsorbs progeny radionuclides that are pumped through with a defined air-flow which reflects the very basic function of the adsorption of progeny in the respiratory tract during breathing. Still, many characteristics of the respiratory tract are missing in this very rudimentary mechanical surrogate. It does not account for the breathing cycle and changing air-flow velocities in combination with the geometry of the respiratory tract. Instead, all radon progeny nuclides are deposited on the filter system. The filter system was exposed within an in-house designed radon chamber^23^ at different controllable radon activity concentrations for one hour, during which the radon activity concentration, relative humidity, temperature and air pressure were recorded. Afterwards, the activity of the γ-emitting isotopes ^214^Pb and ^214^Bi on the filter system was measured until the background level was roughly reached again. The time dependent activity curves allowed us to determine the amount of deposited progeny on the filter system and correlate it to the radon activity concentration and other impacting factors during exposure (e.g. ventilation rate of filters and wall attachment of progeny). Using a set of differential equations, the distribution and dynamical evolution of all decay products in the radon chamber were analysed including the concentration of progeny in the atmosphere, the attachment on the air-flowed filter system and the attachment on the chamber walls. To further verify the mathematical model, activity measurements with a filter system attached to the chamber walls were conducted to independently measure the wall attachment and compare it to the results suggested by the measurements of the air-flowed filter system.

The model allows to consistently describe and understand the experimentally gained data for progeny deposition on an air-flowed filter system in a fully characterized closed system. Here, all radon progeny nuclides are sampled together on the filter system. Therefore, overall energy deposition (given in Gy) on the filters instead of locally applied doses, which would depend on activity size distribution, the respiratory tract morphology and the radiation sensitivity of different cell types, is considered throughout the manuscript.

The model is a first step for experimentally measuring deposition of radon progeny under varying exposure conditions in the respiratory tract and, in a next stage of development, may complement theoretical simulations. So far, this manuscript deals with overall energy deposition on an air-flowed filter system and the comprehensive characterization of the exposure system. Employing aerosol filters to sample radon progeny is the current gold standard^24^, but via the complete mathematical description of our experimental system, we have access to all related quantities. This allows us to also determine the time-dependent distribution of radon progeny radionuclides in the experimental set-up (not only filter but also on the chamber walls and in the atmosphere) and thus a rigorous analysis of the dosimetric data. Therefore, we can determine what part of radon in air arrives on the filter system and how it depends on impacting factors.

## Methods

### Experimental set-up

**Figure 1:**
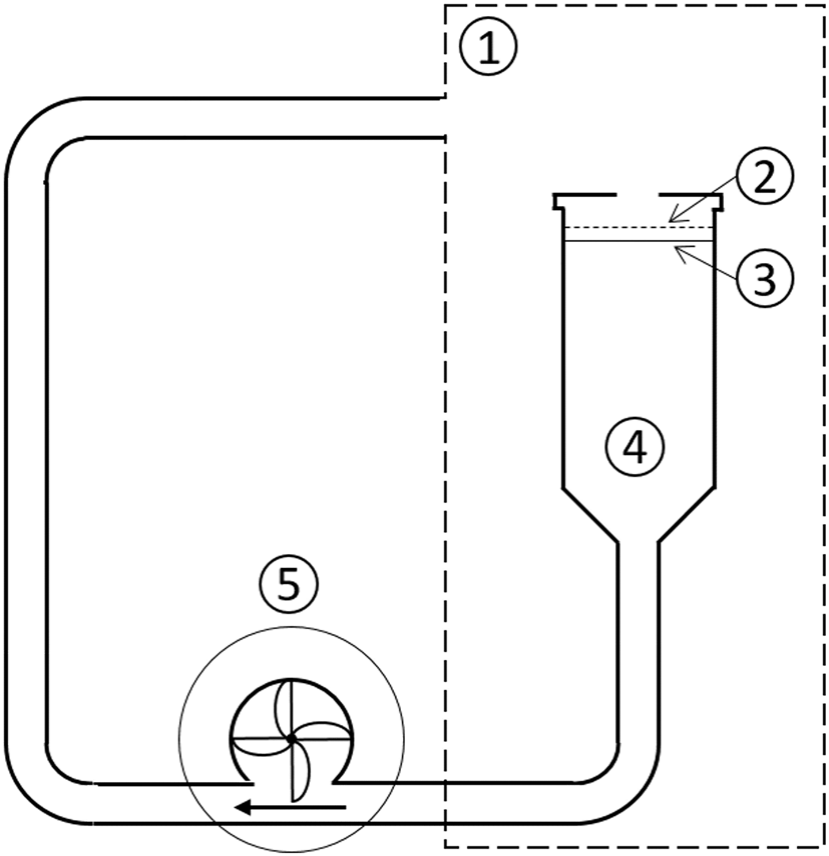
Schematic drawing of the used set-up (not to scale) with: 1 radon chamber, 2 mesh grid, 3 glass fibre filter, 4 centrifuge tube, 5 pump; the arrow shows the direction of flow.

An air-flowed filter system was exposed in an in-house constructed radon chamber. The filter system consists of a 200 mesh stainless steel grid with a wire strength of 0.053 mm (Zivipf.de, Treuchtlingen, Germany) positioned over a 0.4 mm thick glass fibre filter with a pore size of 500 nm (MN85/90, Macherey–Nagel GmbH & Co. KG, Düren, Germany). The two filters have a radius of 27.2 mm each and are separated by a thin wire with a diameter of 0.8 mm. The system was positioned at the upper end of a 50 ml centrifuge tube (Sarstedt AG & Co. KG, Nümbrecht, Germany), on whose lower end a 6 mm wide hole was drilled to connect it to a pump (Micro-Membranförderpumpe NMP 05 B, KNF Neuberger GmbH, Freiburg, Germany) via silicon tubes (Deutsch and Neumann GmbH, Berlin, Germany). The pump produced a defined air-flow of 193.4 ± 2.8 ml min^−1^ from the upper to the lower end of the centrifuge tube through the filter system, which is in the same order of magnitude like the respiratory minute volume of a mouse. A schematic drawing of the set-up is shown in Fig. 1.

The prepared centrifuge tube was placed in a rack in the cylindrical radon chamber that has a height of 14.6 cm and radius of 8.5 cm. Afterwards, the chamber was sealed and the voltage applied to the pump, so that air was pumped through the system and recycled in the chamber. In parallel, the chamber was once flushed with 0.7 l of filtered ^222^Rn containing air, so that pure radon gas was induced in the otherwise sealed chamber. The filter system was exposed for 1 h at constant radon activity concentrations at a preselected level between 0.3 and 6 MBq m^−3^. For experiments with a lower relative humidity during exposure, the radon chamber was flushed for 15 min with dry compressed air before the radon was introduced and the pump was activated. The radon activity concentration and relative humidity in the chamber were tracked during the time of exposure (RTM 1688–2, Sarad GmbH, Dresden, Germany).

At the end of the exposure, the radon chamber was flushed with fresh air for 5 min and the filter system (mesh and glass fibre filter) was removed from the centrifuge tube to record γ-spectra of ^214^Pb and ^214^Bi at different time points after exposure with a high purity Ge-detector (BE3825 Mirion Technologies (Canberra) GmbH, Rüsselsheim, Germany). Therefore, the filters were placed in a petri dish (ø 6.0 cm) to avoid surface contamination of the detector. Afterwards γ-spectra were recorded for up to 4.5 h after end of exposure with varying measurement intervals between 2 and 20 min, depending on the γ-activity.

For an additional consistency check of the mathematical model that fully describes the closed exposure system, filter measurements were conducted at the chamber walls. For this purpose, the same filter system that was placed in the centrifuge tube but with a larger diameter of 55 mm was stuck to the bottom, side wall and lid of the radon chamber during exposure. Afterwards, the filters were removed from the chamber and the γ-activity of ^214^Pb and ^214^Bi was simultaneously measured for all filters with the same detector as for the air-flowed filter system. From this, an average value for the attachment of progeny to the chamber walls was determined.

In total, 13 experiments were conducted at defined radon activity concentrations, thereof 11 with a high relative humidity in the range between 57 and 75% and 2 at lower relative humidity of ~ 20%. In three cases the aforementioned wall exposure was measured in parallel.

### Evaluation of γ-spectra

To determine the time-dependent course of activities of ^214^Pb and ^214^Bi on the exposed filter systems, the area underneath the peaks (at 295 keV and 352 keV for ^214^Pb and at 609 keV for ^214^Bi) in the γ-spectra were determined using the Genie 2000 peak analyses software (Mirion Technologies GmbH, Rüsselsheim, Germany). Afterwards, these areas were background adjusted (step continuum substraction with 12 continuum channels) to get the net peak area N_n,_ which corresponds to the number of decays. Then, the activity $A$ was calculated using the following equation:

1 $$A=\frac{{N}_{\mathrm{n}}{\cdot f}_{1}{\cdot f}_{2}}{\varepsilon \cdot p\cdot T}.$$

Thereby, ${f}_{1}$ is the decay correction taking into account the radioactive decay during measurement time, ${f}_{2}$ the pile-up correction, $\varepsilon$ the efficiency factor including the sample geometry and detector efficiency, $p$ the branching ratio and $T$ the recording time of the spectrum. The detector efficiency was determined by the Genie2000 Software ISOCS/LabSOCS, considering the shape and density of the filter-materials. For the geometry of the filter system a cylinder geometry with a radius of 13.6 mm, a height of 0.7 mm, a density of 0.9946 g cm^−3^ and an absorber (petri-dish, polysterol) with the height of 1 mm was assumed. The same accounts for the pile-up correction, which was negligibly small for all recorded spectra (${f}_{2}\simeq 1$). Additional measurements with calibration sources validated the values obtained by the programme. The decay correction ${f}_{1}$ was determined by an iterative process, in which the developed model was fitted to the experimentally gained data. It is given by the curve shape with

2 $${f}_{1}=\frac{T\cdot A({t}_{i})}{{\int }_{{t}_{i}}^{{t}_{i}+T}A(t)dt},$$

whereby ${t}_{i}$ is the time when the recording started. Then, the recorded activity values are corrected for that factor and the fit procedure is repeated. After the 3^rd^ iteration, values change only marginal so that the procedure is stopped then.

The error bars of the activity values for a single spectrum were determined by Gaussian error propagation from the uncertainties of ${N}_{\mathrm{n}}$ and $\varepsilon$ determined by the Genie 2000 software.

### Mathematical model to describe the experimentally gained data

For the mathematical description of the whole experimental set-up, the exposure system is divided into three components: the air-flowed filter system, the chamber walls and the radon containing atmosphere within the radon chamber. The number of ^218^Po (${N}_{\mathrm{Po}}$), ^214^Pb (${N}_{\mathrm{Pb}}$) and ^214^Bi (${N}_{\mathrm{Bi}}$) atoms during exposure can be described by the following set of differential equations for the filter system marked with the index *f*^24^

3 $$\begin{gathered} \frac{{dN_{{{\text{Po}},{\text{f}}}} }}{dt} = qkN_{{{\text{Po}},{\text{a}}}} - \lambda_{{{\text{Po}}}} N_{{{\text{Po}},{\text{f}}}} \hfill \\ \frac{{dN_{{{\text{Pb}},{\text{f}}}} }}{dt} = qkN_{{{\text{Pb}},{\text{a}}}} + \lambda_{{{\text{Po}}}} N_{{{\text{Po}},{\text{f}}}} - \lambda_{{{\text{Pb}}}} N_{{{\text{Pb}},{\text{f}}}} \hfill \\ \frac{{dN_{{{\text{Bi}},{\text{f}}}} }}{dt} = qkN_{{{\text{Bi}},{\text{a}}}} + \lambda_{{{\text{Pb}}}} N_{{{\text{Pb}},{\text{f}}}} - \lambda_{{{\text{Bi}}}} N_{{{\text{Bi}},{\text{f}}}} , \hfill \\ \end{gathered}$$

the chamber walls marked with the index $w$

4 $$\begin{gathered} \frac{{dN_{{{\text{Po}},{\text{w}}}} }}{dt} = \lambda_{{\text{F}}} N_{{{\text{Po}},{\text{a}}}} - \lambda_{{{\text{Po}}}} N_{{{\text{Po}},{\text{w}}}} \hfill \\ \frac{{dN_{{{\text{Pb}},{\text{w}}}} }}{dt} = \lambda_{{\text{F}}} N_{{{\text{Pb}},{\text{a}}}} + \lambda_{{{\text{Po}}}} N_{{{\text{Po}},{\text{w}}}} - \lambda_{{{\text{Pb}}}} N_{{{\text{Pb}},{\text{w}}}} \hfill \\ \frac{{dN_{{{\text{Bi}},{\text{ w}}}} }}{dt} = \lambda_{{\text{F}}} N_{{{\text{Bi}},{\text{a}}}} + \lambda_{{{\text{Pb}}}} N_{{{\text{Pb}},{\text{w}}}} - \lambda_{{{\text{Bi}}}} N_{{{\text{Bi}},{\text{w}}}} \hfill \\ \end{gathered}$$

and the atmosphere within the chamber marked with the index $a$

5 $$\begin{gathered} \frac{{dN_{{{\text{Po}},{\text{a}}}} }}{dt} = \lambda_{{{\text{Rn}}}} N_{{{\text{Rn}},{\text{a}}}} - \left( {\lambda_{{\text{F}}} + qk + \lambda_{{{\text{Po}}}} } \right)N_{{{\text{Po}},{\text{a}}}} \hfill \\ \frac{{dN_{{\text{Pb,a}}} }}{dt} = \lambda_{{{\text{Po}}}} N_{{\text{Po,a}}} - \left( {\lambda_{{\text{F}}} + qk + \lambda_{{{\text{Pb}}}} } \right)N_{{\text{Pb,a}}} \hfill \\ \frac{{dN_{{{\text{Bi}},{\text{a}}}} }}{dt} = \lambda_{{{\text{Pb}}}} N_{{{\text{Pb}},{\text{a}}}} - (\lambda_{{\text{F}}} + qk + \lambda_{{{\text{Bi}}}} )N_{{{\text{Bi}},{\text{a}}}} . \hfill \\ \end{gathered}$$

In these equations the first term on the right-hand side reflects the replenishment of the isotopes from the chamber atmosphere. The last term accounts for further decay at the considered compartment, therefore appearing negative. For the filter and walls, the equations for Pb and Bi contain a third (middle) term, reflecting the decay of previously adhered mother nuclides. For the atmosphere, the isotopes may attach to the chamber wall (${\lambda }_{\mathrm{F}}$) and the air-flowed filter system ($qk$), so that their concentration in the atmosphere is reduced. The quantities ${\lambda }_{\mathrm{Rn}},{ \lambda }_{\mathrm{Po}}, {\lambda }_{\mathrm{Pb}}$ and ${\lambda }_{\mathrm{Bi}}$ represent the decay constants of the associated nuclide. The relative flow rate $q$, i.e. the fraction of the chamber volume (3.31 l) which passes the filter system per time, is given with (9.74 ± 0.14)·10^−4^ s^−1^. Additionally, $k$ gives the sticking probability for the progeny radionuclides that pass the filter system, which is practically given by 1. Because pure radon gas without progeny is introduced to the chamber the majority of the decay products is not attached to larger aerosols and show small diameters in the range of several nm, so that their diffusion coefficient is high. Thus, there is a high probability that they will adhere to the surface of the filter. But also progeny radionuclides that attach to aerosols during the time of exposure, thus showing larger diameters, will mainly be adsorbed by the glass fibre filter (500 nm pore size). Also, additional measurements with two filters in a row could confirm that this choice of $k$ is justified.

The parameter ${\lambda }_{\mathrm{F}}$ is the only free fit parameter and accounts for the wall attachment of the progeny radionuclides, so that they get attached to the chamber walls with a half-life of ${t}_{1/2\mathrm{F}}=\frac{\mathrm{ln}2}{{\lambda }_{\mathrm{F}}}$. Initial conditions to solve the differential equations are given with ${N}_{\mathrm{Rn}}\left(t=0\right)=\frac{{c}_{\mathrm{Rn}}\cdot {V}_{\mathrm{chamber}}}{{\lambda }_{\mathrm{Rn}}}, {N}_{\mathrm{Po}}\left(t=0\right)={N}_{\mathrm{Pb}}\left(t=0\right)={N}_{\mathrm{Bi}}\left(t=0\right)=0$, where ${c}_{\mathrm{Rn}}$ is the preselected radon activity concentration during exposure and ${V}_{\mathrm{chamber}}$ the chamber volume of 3.31 l. After 60 min of exposure the air-flowed filter system is removed from the radon containing atmosphere. The change of atoms for ^218^Po, ^214^Pb and ^214^Bi can then be described by an adapted set of Eq. (3) without the first term on the right-hand side, as the replenishment of isotopes ($q$) from the chamber air is stopped. The number of each nuclide at the start of the evaluation time is given by the solution for the number of nuclides after 60 min in Eq. (3). By multiplying the course of the particle number of each nuclide with its decay constant, we obtained its course of activity on the filter system.

To describe the activity measurements on the filter system fixed at the chamber walls, the set of differential equation is the same as for the surface of the chamber but scaled to the smaller area of the filters that were measured (index wf). The scaling factor is the ratio of the two areas given by $u=\frac{3\pi {r}_{\mathrm{wf}}^{2}}{2\pi {r}_{\mathrm{chamber}}({r}_{\mathrm{chamber}}+{h}_{\mathrm{chamber}})}$ with $r_{\mathrm{wf}}$ being the radius of the wall filter, $r_{\mathrm{chamber}}$ the radius of the cylindrical chamber, $h_{\mathrm{chamber}}$ its height and 3 the number of used wall filters. The set of differential equations for the filters on the wall after exposure is the same as for the air-flowed filter system. They only differ by their initial conditions because the number of nuclides on the wall after 60 min exposure from Eq. (4) scaled with $u$ is used for the wall filters. Again, the multiplication of the obtained curves with the decay constant of the associated nuclide gives the course of its activity.

This model can now be fitted to the experimentally obtained activity data of ^214^Pb and ^214^Bi recorded for single experiments with different radon activity concentrations in the chamber. Assuming a linear relation between the radon activity in the radon chamber and the activity on the filter system, all data were linearly scaled to a reference activity value of 1 MBq m^−3^ to allow for a joint analysis and to obtain one representative average constant for the wall attachment. The error of the scaled activities is given by Gaussian error propagation concerning the uncertainty of the radon activity concentration given by the accuracy of the measurement device and the uncertainties for the non-scaled activities from the single spectra (see Eq. (1)).

To investigate the relation between the radon activity concentration during exposure and the activity on the filter system (see Fig. 3), all non-scaled data sets were also fitted. The retrieved activities on the filter system directly after exposure (initial activities) were plotted against the radon activity concentration during exposure. The error for the initial activities are gained by Gaussian error propagation considering the uncertainty of the air-flow through the filter system ($\Delta q$) the wall attachment constant ($\Delta {\lambda }_{F}$) from the fit of the single experiments. Uncertainties concerning the radon activity concentration during an experiment are originating from the accuracy of the measurement device. Additionally, the linear extrapolation of the initial activities from the joint analysis of all experiments is plotted in Fig. 3 (straight lines). To derive it, the initial activity for each nuclide from the joint analysis of all data scaled to 1 MBq m^−3^ is used and linearly rescaled to the actual radon activity concentrations in the different experiments. Also, the error of this initial activity (originating from $\Delta q$ and $\Delta {\lambda }_{F}$ from the joint analysis) is linearly scaled and represented by the error bands.

### Calculation of deposited energy on the air-flowed filter system

By applying the model to the experimental data for ^214^Pb and ^214^Bi, activity curves for the radon progeny ^218^Po, ^214^Pb and ^214^Bi were obtained. The integral of the activity curves gives the total number of decays for each isotope.

Notably, the activity of ^214^Pb, which is determined by the area underneath the ^214^Pb curve, consists of two components. The first component is the ^214^Pb produced by decaying ^218^Po, which is already on the filter system. The second component is ^214^Pb, which is produced from ^218^Po-decay in the atmosphere and subsequently deposited on the filter during the one-hour exposure. The same accounts for ^214^Bi. Due to its short half-life of 164 μs, its daughter nuclide ^214^Po can be expected to be in radioactive equilibrium with ^214^Bi almost immediately.

For the estimation of the total deposited energy, only the α-particle emissions are regarded to be relevant, releasing α-energies of 6.0 MeV and 7.69 MeV for ^218^Po and ^214^Po, respectively. For the determination of the deposited energy on the filter system, the determined number of decays from ^218^Po can be directly multiplied with its potential alpha-energy (see Table 1). This already accounts for the energy release from all further decaying daughter nuclides on the filter system. To add contributions from the decay products ^214^Pb and ^214^Bi, which readily attached to the filter, the area of the activity curves of the respective mother nuclide must be subtracted. Thereby quantified is the amount of nuclides which are produced during the one-hour exposure in the chamber atmosphere and subsequently deposit on the filter. These numbers are then also multiplied with their respective potential α-energy. By addition of all three components, the deposited energy is obtained.

**Table 1:** Emitted and potential alpha-energy for radon decay products.

Nuclide	^218^Po	^214^Pb	^214^Bi	^214^Po
Emitted α-energy (MeV)	6.00			7.69
Potential α-energy (MeV)	13.69	7.69	7.69	7.69

### Ethics and inclusion statement

No human patients were involved during this study.

## Results

### Time-dependent activity of radon progeny

**Figure 2:**
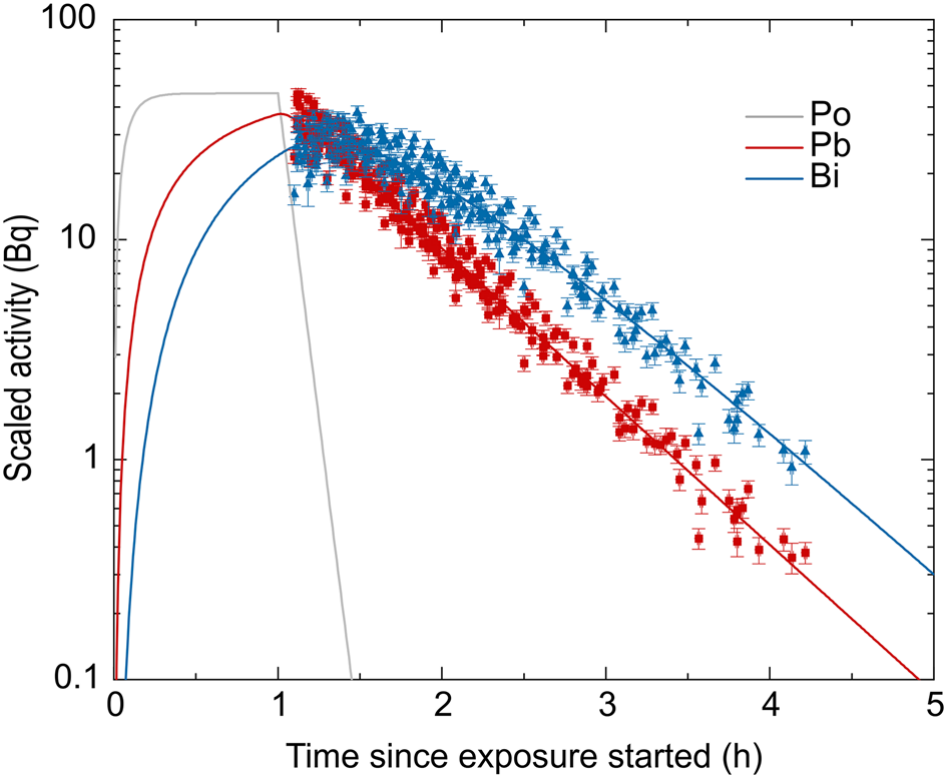
Experimentally determined, decay-corrected activities of ^214^Pb (red) and ^214^Bi (blue) for the air-flowed filter system, that were scaled for a reference activity concentration of 1 MBq m^−3^. The fitted course of ^218^Po- (grey), ^214^Pb- and ^214^Bi-activities was determined with the model describing distribution and decay of the relevant nuclides and revealed a constant for wall attachment of ${\lambda }_{F} = 3.890 \pm 0.033$ min^−1^ (number of experiments: N = 13). Error bars are gained with Gaussian error propagation from the uncertainty of the radon activity concentration (accuracy of measurement device) and non-scaled activities (see section “Evaluation of γ-spectra”) but do not account for non-captured differences between different experiments (e.g. $\Delta q$ and Δ﻿$\Delta \lambda_F$).

After a one-hour exposure of the filter system in the radon chamber at constant, preselected radon activity concentrations ranging from 0.6-5.5 MBq m^−3^, the energy spectra of the γ-emitting isotopes ^214^Pb and ^214^Bi were measured for up to 4.5 h after exposure. The air-flow rate through the filter system and exposure time were the same for all measurements but the radon activity concentrations varied between the 13 individual experiments. Therefore, all activity values were linearly scaled to a radon reference activity concentration of 1 MBq m^−3^, allowing data pooling. These pooled activity values and the fit based on the quantitative model description for the activity on the filter (see "Methods" section) are shown in Fig. 2.

The data evolution for all experiments (N = 13) is in good agreement after the scaling. In the first hour during the exposure, the activities of all nuclides rise, whereby ^218^Po approximates a constant value due to the equilibrium between attachment and decay on the filter system. The isotopes ^214^Pb and ^214^Bi do not reach the equilibrium during exposure due to their longer half-life. After exposure, no new progeny radionuclides get attached on the filter system and the accumulated attached progeny decays further, so that the ^218^Po activity decreases exponentially. Also, the ^214^Pb activity decreases exponentially, whereby a very short delay is observed due to the replenishment of more ^214^Pb from the decay of the mother nuclide ^218^Po.

A more pronounced delay and even a built-up in activity is observed for ^214^Bi because of the decay of ^214^Pb. This delay phenomenon is more distinct for ^214^Bi than for ^214^Pb as the half-life of the corresponding mother nuclide, ^218^Po and ^214^Pb, respectively, is longer for ^214^Bi than for ^214^Pb. After about 4 h most of the ^214^Pb has decayed, so that only a small amount of ^214^Bi is delivered and the ^214^Bi activities also approximate an exponential decay. The measured activities of ^214^Pb and ^214^Bi are adequately described by the developed fit model. In particular the model describes the shape of the activity course well, justifying that the model henceforth can be used to describe the experimentally gained data. As the air-flow rate through the filter system just as the radon activity concentration during exposure are known and the amount of progeny at the beginning of exposure is negligibly small, the only free fit parameter of the model is given by the wall attachment rate ${\lambda }_{\mathrm{F}}$(see Method section). The joint fit of all data in Fig. 2 gives a half-life of 10.7 ± 0.1 s for the average wall attachment (i.e. after this time half of the progeny radionuclides have attached to the walls), and thus an attachment constant of ${\lambda }_{\mathrm{F}}=$ 3.890 ± 0.033 min^−1^ is representative for all experiments.

Nevertheless, fluctuations of the scaled activities exceed their error. For one experiment the calculated errors explain about 70% of the entire fluctuations, but the errors of the scaled activities do not account for additional factors that change between the experiments with different radon activity concentration and have an impact on the wall attachment constant ${\lambda }_{F}$ (like humidity, different aerosol size distribution etc.). This shows that the radon activity concentration is not the only parameter that influences the amount of progeny on the filter system, but other factors in the chamber atmosphere also influence progeny deposition. The impact of these non-captured uncertainties can be assessed by considering the fluctuations of data in Fig. 2 and will be further analysed in the following section. Therefore, the price of a joint examination of all experiments is combining measurements of different settings, where comparative uncertainty sources are not fully known (e.g. fluctuations in ${\lambda }_{F}$ between different experiments).

### Progeny activity in dependence of ambient radon activity concentration

**Figure 3:**
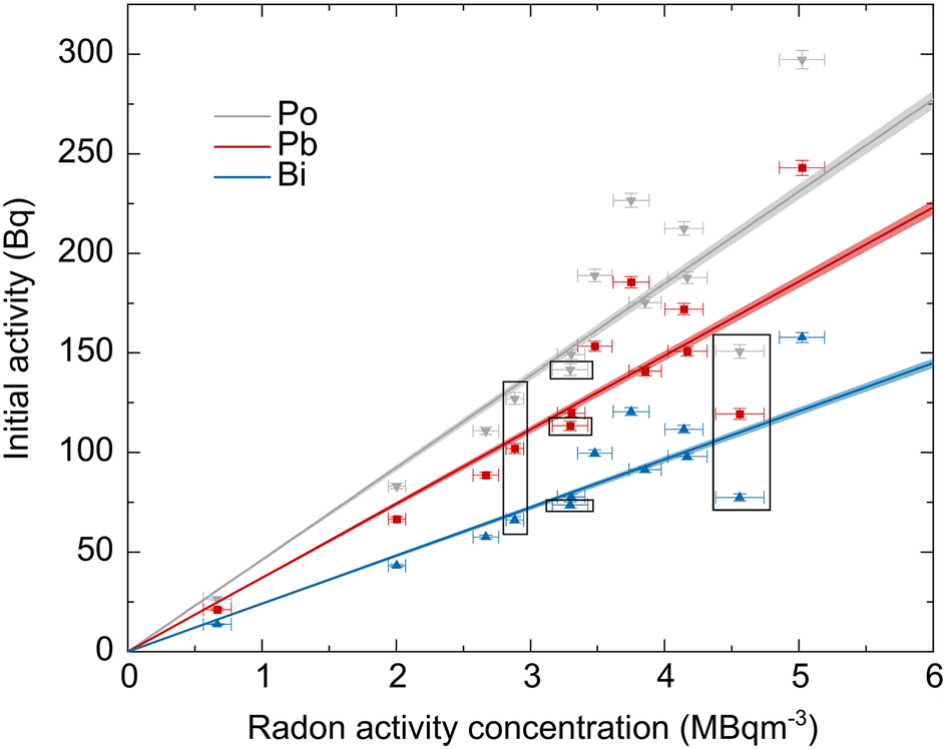
From the non-scaled data sets determined initial activities of ^218^Po (grey), ^214^Pb (red) and ^214^Bi (blue) for the air-flowed filter system plotted against the radon activity concentration during exposure. Errors in vertical direction are gained with Gaussian error propagation with respect to the uncertainty of the parameters in Eqs. (3–5) (uncertainty for air-flow and the wall attachment constant from single experiments), uncertainties concerning the radon activity concentration are originating from the measurement device. The straight lines represent the linearly rescaled initial activity for each nuclide determined from the joint analysis of all experiments (activities scaled to 1 MBq m^−3^). The error margin is also linearly scaled and originates from the uncertainty of the initial activities, which is calculated with Gaussian error propagation from the uncertainty for air-flow and the wall attachment constant from the joint analysis. Therefore, the error margin accounts for the uncertainty of the best fit, but not for the fluctuation margin from single experiments. Framed data are data points gained at lower relative humidity during exposure.

In the previous analysis, all filter measurements from different experiments were scaled to a reference radon activity concentration of 1 MBq m^−3^ during exposure and pooled afterwards. In this section the unscaled measurements are considered and we analyse the relation between the amount of attached progeny on the air-flowed filter system and the ambient radon activity concentration in the radon chamber during exposure in more detail. Therefore, the mathematical description of the measured activity values of ^214^Pb and ^214^Bi was fitted individually to each non-scaled data-set. From these fits the activities of ^218^Po, ^214^Pb and ^214^Bi right after exposure (in the following called initial activity) on the air-flowed filter system were extracted and plotted against the independently measured radon activity concentration (see “Methods” section). The result is shown in Fig. 3.

In addition, the initial activity for ^218^Po, ^214^Pb and ^214^Bi derived from the pooled analysis were rescaled to the actual radon activity concentration during exposure, which is represented by the straight lines in Fig. 3. By comparing the rescaled activity with the individually determined activities from each fitted data-set, the expected increase of the amount of progeny on the air-flowed filter system with the radon activity during exposure is confirmed, while the data show fluctuations and some overproportional increase added to a linear relationship. This result indicates a lower wall attachment constant at higher radon activity concentrations.

Moreover, fluctuations of the initial activities from the single experiments become stronger for higher radon activity concentrations during exposure, so that significant deviations from the rescaled values of the joint analysis (straight lines) are observed for radon activity concentrations higher than 4 MBq m^−3^. This indicates a stronger influence of the wall attachment constant at higher radon activity concentrations.

Especially striking is the low activity detected for all nuclides at a radon activity concentration of 4.56 ± 0.18 MBq m^−3^ during exposure (one of the framed values in Fig. 3). During this experiment, a quite low relative humidity of 22.1 ± 0.1% was present during exposure in comparison to the normally predominant ~ 70%. Two additional experiments at lower relative humidity (20.2 ± 0.1% and 57.3 ± 0.1%, see framed data points in Fig. 3) were conducted to investigate the influence of the air humidity on the amount of progeny on the air-flowed filter system. A reduced number of aerosols in air would lead to more unattached progeny with a smaller size than the size of the attached progeny^25^. The smaller the particles are, the higher is their diffusion coefficient and thus the attachment to walls due to diffusion-driven processes. This could lead to a higher wall deposition and thus to a smaller amount of progeny on the air-flowed filter system. But this effect seems to become only relevant at higher radon activity concentrations as no strong influence was shown in the measurements with low vs high relative humidity during exposure at lower radon activity concentrations.

### Wall attachment of radon progeny

**Figure 4:**
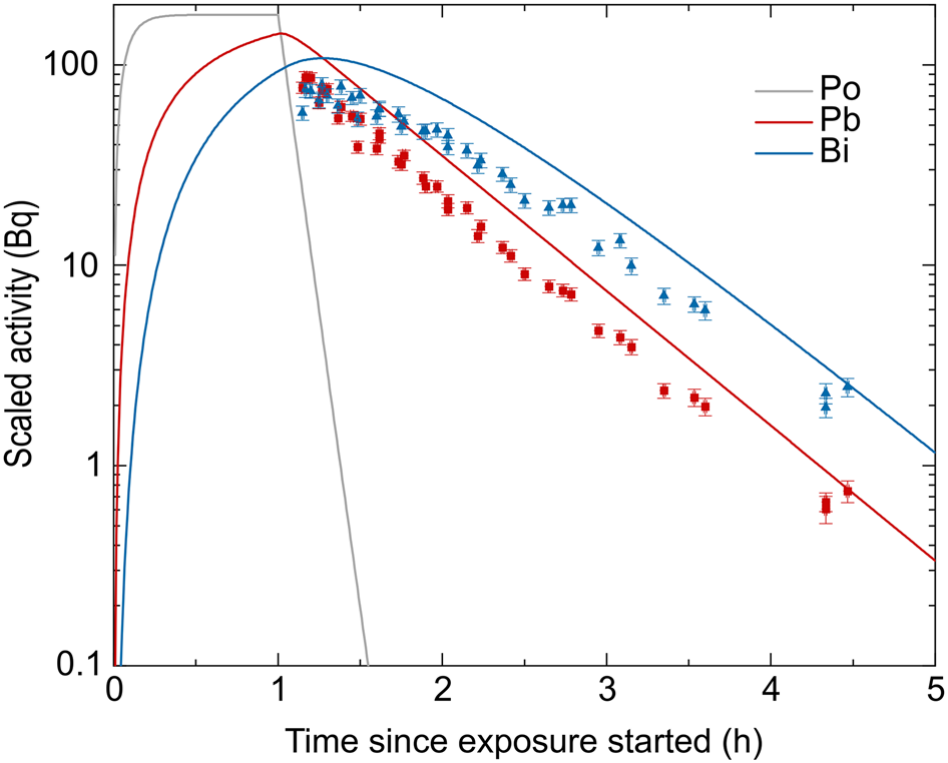
Experimentally determined, decay-corrected activities of ^214^Pb (red) and ^214^Bi (blue) on the wall filter system attached to the radon chamber walls, scaled according to an ambient reference Rn concentration of 1 MBq m^−3^(data points). Error bars are gained by the error of the single parameters used for activity determination with Gaussian error propagation. The calculated course of expected ^218^Po- (grey), ^214^Pb- (red) and ^214^Bi-(blue) activities derived from the model approach with the constant for wall attachment from the air-flowed filter system given with ${\lambda }_{F} = 3.890 \pm 0.033$ min^−1^ are shown in comparison (lines).

To further verify our mechanistic understanding, the same filter system that was used for the flow measurements, was attached to the bottom, side wall and lid of the radon chamber and exposed for one hour to determine the activity loss by attachment to the chamber walls. The filter was removed after exposure and the activities of ^214^Pb and ^214^Bi were recorded for up to 4.5 h in the same way as for the flow measurements. Afterwards, all activity values were linearly scaled to a radon activity concentration of 1 MBq m^−3^ (see Fig. 4) as before for the activities on the air-flowed filter system. By using the attachment constant of ${\lambda }_{\mathrm{F}}=$ 3.890 ± 0.033 min^−1^ from the pooled analysis of the air-flowed filter system (see section "Time-dependent activity of radon progeny"), the independently calculated expected course of activity from the developed mathematical model on the filter system mounted to the chamber walls is represented as well in Fig. 4.

The general course of the expected ^214^Pb and ^214^Bi activities from our model on the wall-attached filter system reveals the same shape and is in good agreement with the actual measurement. The calculated and therefore expected activity values exceed the actual measured ones by a factor 1.6. This has two reasons: first, the surface on which the progeny in the exposure system can attach is larger than assumed by the scaling factor in the mathematical model (see methods section). In this model only the inner surface of the radon camber is accounted for, but not the surfaces of all items of the experimental set-up, including the mount of the filter system and various tubes. Therefore, the amount of progeny that attaches to the surface is estimated from a smaller boundary surface than is actually present and thus results in a higher calculated surface activity, i.e. an overestimation of activity per wall area. Second, the filter system was attached to smooth areas of the walls, so that edges of the chamber, where the progeny radionuclides tend to accumulate in higher concentration, were not accounted for in the wall measurements. Therefore, the measured activities with the wall-attached filter system show a lower amount of progeny than is actually expected including the edges. As we did not account for these factors in our model, the discrepancies reflect sources of expected model uncertainties, apart from which the model prediction agree fairly well with the actual measured activity curves of ^214^Pb and ^214^Bi on both the walls and the air-flowed filter.

### Energy determination on the air-flowed filter system

To determine the energy deposited on the air-flowed filter system, first the integral of the activity curves, which represents the number of decays of the corresponding nuclide, are calculated from the model fit. Here, the area under the ^214^Bi curve is the largest as it contains ^214^Bi decays following the decaying ^218^Po and ^214^Pb, which is produced on the filter in addition to the decays of ^214^Bi nuclides directly deposited from the chamber atmosphere. Still, the area underneath the ^214^Pb curve is just marginally smaller. This is reasoned as most of the ^222^Rn progenies ^218^Po and ^214^Pb are already deposited to the chamber walls and filter system before their decay, so that only a very small amount of ^214^Bi atoms is present in the chamber atmosphere and directly attaches to the filter system. The area underneath the ^214^Pb curve reflects ^218^Po nuclides that have decayed while already being adherent to the filter and the directly deposited ^214^Pb from the chamber atmosphere. Finally, the area underneath the ^218^Po curve is the smallest, consisting solely of directly deposited polonium.

For the reference activity concentration of 1 MBq m^−3^ and 1 h exposure time the activity integrals can be used to determine the corresponding energy depositions, allowing for further dosimetric considerations. By multiplying the numbers of directly deposited nuclides with their potential alpha energy ${\varepsilon }_{i}$ (see methods section), the deposited energy ${E}_{i}$ for each isotope $i$ can be calculated (see Table 2).

6 $${E}_{i}={\int }_{0}^{\infty }{A}_{i}(t){\varepsilon }_{i}dt-{\int }_{0}^{\infty }{A}_{i-1}(t){\varepsilon }_{i-1}dt,$$

whereby ${A}_{i}$ denotes the scaled activity over time (see Fig. 2) and the index $i-1$ refers to the mother nuclide of $i$. For ^218^Po the second part of the equation is given by 0 because it is the first progeny radionuclide in the decay chain.

**Table 2:** Deposited energy in the filter system for separate radon decay products for the reference activity concentration of 1 MBqm^−3^ and 1 h exposure time.

Nuclide	^218^Po	^214^Pb	^214^Bi
Deposited energy (GeV)	2268.58 ± 17.72	72.57 ± 1.16	0.47 ± 0.01

Notably the contributions of directly deposited nuclides get rapidly smaller along the decay chain. Therefore and due to its very small half-life further contributions of ^214^Po can be neglected. In sum, a total deposited energy of about 2341 GeV is determined.

## Discussion

Here we presented a prototype of an experimental mechanical surrogate system to simulate how the overall deposition of radon progeny in the respiratory tract depend on external environmental factors. We developed a mathematical model to describe the deposition of radon progeny on an air-flowed filter system, which is placed inside a radon chamber. Besides the progeny attachment on the filter, the model includes the deposition on the chamber surface and the radioactive decay of each nuclide.

By investigating the general characteristics of radon progeny deposition, we examined the relation between the radon activity concentration in ambient air and the amount of captured progeny on the air-flowed filter system. Notably, the latter is proportional to dose. In general, the amount of deposited progeny seems to rise almost linearly with a slightly increasing slope with the radon activity concentration. Nevertheless, strong fluctuations with significant deviations from a linear relation are only observed for radon activity concentrations higher than 4 MBq m^−3^, so that the wall attachment constant tends to higher variability at higher radon activity concentrations. Also, the influence of changing relative humidity on the wall attachment seems to be higher at higher radon activity concentrations without an apparent explanation, making further investigations necessary (see Fig. 3). As stronger fluctuations in the relation between the deposited progeny and ambient radon activity concentration are only observed for higher radon activity concentrations, we can translate conclusions from the linear relations obtained from all experiments (lines in Fig. 3 which are close to experimental data at lower concentrations) to real scenarios without a severe impact of fluctuations.

The worldwide average indoor radon activity concentration was estimated as 39 Bq m^−3^ with huge regional and temporal fluctuations in the order of several kBq m^−3^^26^. Consequently, for radon exposure in every-day life, it can be hypothesized, that exposure to higher radon activity concentrations leads to proportionally higher doses in the respiratory tract if all other exposure conditions (e.g. breathing rate and aerosol size distribution) stay the same. This assumption proofs consistency with current radiation protection models and we can verify this fact with our simple model, which is less affected by internal or external factors than human or animal studies.

For dosimetry in a real respiratory tract or a mechanical surrogate system including bifurcating airways, in addition to the ambient activity concentration also aerosol size distribution and breathing rate play an important role, as this determines the location of energy deposition. Inhaled aerosols can deposit in the respiratory tract via three distinct mechanisms (inertial impaction, gravitational sedimentation and Brownian motion)^21,25,26^ and stay there during exhalation^18^. Only the consideration of these processes would allow for a detailed model of dosimetry in the airways^14^. Moreover, not only the respiratory minute volume determines the number of deposited particles in the respiratory tract like assumed in our model, but also the breathing rate, because it determines, where particles are deposited and which amount will be exhaled again.

But already from the findings in the present study, in which we consider the respiratory tract as one compartment in which all inhaled progenies are deposited, dose values for the respiratory tract of a mouse can be estimated. Therefore, the parameter of the air-flowed filter system are adapted to anatomical values of a mouse. The obtained dose values can then be compared to the literature.

Based on our experimental and mathematical model, different parameters that influence the applied dose to the whole respiratory tract following exposure can be investigated. The dose $D$ in a mouse lung can be estimated by the equation

7 $$D=\frac{{E}_{exp}\cdot \frac{{f}_{ml}}{{f}_{exp}}}{{m}_{ml}}$$

with the deposited energy on the experimental filter system ${E}_{exp}=2341 \mathrm{GeV}$ for reference conditions (activity concentration of 1 MBq m^−3^ and 1 h exposure time) and the approximate mass of a mouse lung ${m}_{ml}=152 \pm 3$ mg (mean ± standard error of the mean)^27^. To scale the deposited energy from the filter system to a mouse lung, the ratio of the ventilation rates appear in the equation as well, where the rate for mouse lung is just the breathing rate of a mouse ${f}_{ml}=60 \pm 6$ ml min^−1^ (mean ± standard error of the mean)^28^, and the rate for the filter system is the air-flow rate ${f}_{exp}=193.4 \pm 2.8$ ml min^−1^. Evaluating this equation and normalizing it to the reference conditions mentioned above, a dose rate of 0.76 ± 0.08 nGy (Bq m^−3^)^−1^ h^−1^ is determined. The error was calculated by Gaussian error propagation.

Simulation studies by Sakoda et al. obtained a dose rate for the whole mouse-lung of 34.8 nGy (Bq m^−3^)^−1^ h^−1^ for exposure to radon progeny for conditions similar to mine sites^29^, in which in humans (not mice) only about 12.5% of the inhaled particles are deposited in the lung and excludes the fraction of progeny being exhaled again or deposited in the upper airways^18^. Furthermore, an equilibrium factor of 0.4 is assumed, meaning that the concentration of radon progeny in air is 40% of the concentration expected in equilibrium with radon. In our setup, a deposition of 100% (sticking probability of 1) and an equilibrium factor of around 0.01 was estimated, as pure radon gas is induced in the radon chamber and its progeny radionuclides start to slowly build up during exposure. Although it is only a rough estimate motivated by a measurement of the equilibrium factor in a bigger radon chamber (50 l), the order of magnitude for this factor is well represented by this assumption. When considering this $\frac{0.4}{0.01}\cdot\frac{0.125}{1}=$ fivefold increase, our dose rate is 3.8 nGy (Bq m^−3^)^−1^ h^−1^. Thus, our obtained dose values are slightly lower than given by Sakoda et al., probably due to a slight overestimation of the F-factor from the bigger compared to the smaller chamber. Nevertheless, it should be noted, that this does not account for the inhomogeneous distribution of radon progeny in the airway system, but is only the average dose over the whole lung. It also underlines the importance of the equilibrium factor for radon dosimetry.

The same model simulation for exposure to radon progeny was done by Sakoda et al. for exposure to pure radon gas, where a dose rate of 25.4 pGy (Bq m^−3^)^−1^ h^−1^ was determined. These data are useful for places with a very low equilibrium factor, meaning that the concentration of radon progeny in air is expected to be low^30^.

The here presented model provides the following extensions to currently existing publications:

1. While other publications are only focusing on human exposure^31^ or spatial attachment at different anatomical regions in the lung and their biological effects on cellular level^32,33^, our experimental and mathematical model complement these data.
2. The here presented measurements and models are further validating our method used for dose determination in a voluntary patient after radon therapy^34^, in which a dose in the order of single digit µGy was deduced. In this experiment, the radon activity concentration was 55 kBq m^−3^ and radon progeny radionuclides were filtered. Therefore, the conditions were comparable to the simulations done by Sakoda et al. for pure radon gas^30^ and dose values are in good agreement to these results.
3. Our set-up enables to experimentally investigate different exposure scenarios, e.g. aspects of higher breathing rates during exposure, and thus verify current simulations. This is important as also the ICRP suggests higher applied doses for an increased physical activity and the accompanying higher breathing rate of a person during radon exposure, so that they recommend a dose conversion factor for worker with high physical activity of 20 mSv WLM^−1^ instead of 10 mSv WLM^−1^^18^.

However, it should be noted, that in the exposure scenario assumed by ICRP there is a higher radon progeny concentration and attachment to walls is reducing it negligibly. This is different to our exposure scenario, in which wall attachment is much higher and must be considered.

Considering the 3rd aspect listed above, to simulate the influence of a higher breathing rate, the throughput of air through the exposed filter system could be enhanced and the influence on the amount of attached progeny determined, although the effect of changing airflow velocities in the respiratory tract cannot be investigated with the current model. Nevertheless, our developed experimental setup and theoretical analysis allow the investigation of deposition of radon progeny on a basic level. It could be expanded to a more sophisticated setup in order to distinguish between different deposition mechanisms and more anatomical details as e.g. bifurcation of airways. In consequence, the deposition processes of progeny in the respiratory tract could be traced and used to determine spatially resolved dose distributions and validate the calculated absorbed doses.

This work is the first step for the development of an experimental surrogate system to investigate the deposition of radon progeny under varying exposure conditions in the lung of organisms, applicable to e.g. mice in animal experiments and humans for radiation protection purposes.

## Data Availability

The datasets used and/or analysed during the current study available from the corresponding author on reasonable request.
