# Author Correction: Radon progeny measurements in a ventilated filter system to study respiratory-supported exposure

**DOI:** 10.1038/s41598-023-39546-z

**Published:** 2023-08-01

**Authors:** Franziska Papenfuß, Andreas Maier, Sonja Sternkopf, Claudia Fournier, Gerhard Kraft, Thomas Friedrich

**Affiliations:** 1 GSI Helmholtzzentrum für Schwerionenforschung GmbH, Planckstraße 1, Darmstadt, Germany; 2 Goethe Universität Frankfurt, Max-Von-Laue-Str. 1, Frankfurt, Germany

Correction to: *Scientific Reports* 10.1038/s41598-023-37697-7, published online 04 July 2023.

In the original version of this Article, Gerhard Kraft was incorrectly affiliated with ‘Goethe Universität Frankfurt, Max-Von-Laue-Str. 1, Frankfurt, Germany.’

The correct affiliation is as follows:

1. GSI Helmholtzzentrum für Schwerionenforschung GmbH, Planckstraße 1, Darmstadt, Germany.

The original Article has been corrected.

